# A recombinant multi-antigen vaccine formulation containing *Babesia bovis* merozoite surface antigens MSA-2a_1_, MSA-2b and MSA-2c elicits invasion-inhibitory antibodies and IFN-γ producing cells

**DOI:** 10.1186/s13071-016-1862-1

**Published:** 2016-11-14

**Authors:** Alba Marina Gimenez, Katia S. Françoso, Jonatan Ersching, Marcelo Y. Icimoto, Vitor Oliveira, Anabel E. Rodriguez, Leonhard Schnittger, Monica Florin-Christensen, Mauricio M. Rodrigues, Irene S. Soares

**Affiliations:** 1 CTCMOL, Departamento de Microbiologia, Imunologia e Parasitologia, Universidade Federal de São Paulo-Escola Paulista de Medicina, Rua Mirassol, 207, São Paulo, 04044-010 SP Brazil; 2 Departamento de Análises Clínicas e Toxicológicas, Faculdade de Ciências Farmacêuticas, Universidade de São Paulo, 05508-900 São Paulo, SP Brazil; 3 Departamento de Biofísica, Universidade Federal de São Paulo, CEP 04023-062 São Paulo, Brazil; 4 Instituto de Patobiologia, CICVyA, INTA-Castelar, 1686 Hurlingham, Argentina; 5 CONICET, C1033AAJ Ciudad Autonoma de Buenos Aires, Argentina

**Keywords:** *Babesia bovis*, Merozoites, Recombinant vaccine

## Abstract

**Background:**

*Babesia bovis* is a tick-transmitted protozoan hemoparasite and the causative agent of bovine babesiosis, a potential risk to more than 500 million cattle worldwide. The vaccines currently available are based on attenuated parasites, which are difficult to produce, and are only recommended for use in bovines under one year of age. When used in older animals, these vaccines may cause life-threatening clinical symptoms and eventually death. The development of a multi-subunit recombinant vaccine against *B. bovis* would be attractive from an economic standpoint and, most importantly, could be recommended for animals of any age. In the present study, recombinant ectodomains of MSA-2a_1_, MSA-2b and MSA-2c antigens were expressed in *Pichia pastoris* yeast as secreted soluble peptides.

**Results:**

The antigens were purified to homogeneity, and biochemically and immunologically characterized. A vaccine formulation was obtained by emulsifying a mixture of the three peptides with the adjuvant Montanide ISA 720, which elicited high IgG antibody titers against each of the above antigens. IgG antibodies generated against each MSA-antigen recognized merozoites and significantly inhibited the invasion of bovine erythrocytes. Cellular immune responses were also detected, which were characterized by splenic and lymph node CD4^+^ T cells producing IFN-γ and TNF-α upon stimulation with the antigens MSA-2a_1_ or MSA-2c.

**Conclusions:**

These data strongly suggest the high protective potential of the presented formulation, and we propose that it could be tested in vaccination trials of bovines challenged with *B. bovis*.

**Electronic supplementary material:**

The online version of this article (doi:10.1186/s13071-016-1862-1) contains supplementary material, which is available to authorized users.

## Background

*Babesia bovis* is a protozoan hemoparasite of the phylum Apicomplexa and is the causative agent of bovine babesiosis. In South America, the parasite is transmitted by the common cattle tick (*Rhipicephalus microplus*), and it is distributed mostly in the northeast and northwest of Argentina, in the south-central regions of Brazil, and in other tropical and subtropical regions around the world. From an economic point of view, bovine babesiosis is the most important disease of cattle that is transmitted by arthropods and constitutes a potential risk to more than 500 million cattle [1, 2]. Prospectively, an increased number of cattle will be at risk of bovine babesiosis due to predicted expansion of the vector’s habitat in response to global warming.

In hyperendemic areas, calves become infected at a young age with *B. bovis* parasites and due to protective mechanisms of innate immunity, they do not develop clinical symptoms either upon infection or as adults. Calves infected at a young age remain protected for life. However, if the first exposure to the parasite occurs in animals older than 1 year of age, an immune imbalance is observed, which is characterized by pro-inflammatory cytokine release and the development of serious clinical symptoms, such as high temperature, sensory depression, anemia and uremia. Erythrocytes that are parasitized by *B. bovis* are sequestered in brain capillaries, leading to ischemia and neurological shock. In untreated animals, the infection can rapidly lead to abortions and death [2, 3].

Climatic factors, the erratic use of acaricides, and the development of acaricide resistance can all lead to fluctuations in the populations of *B. bovis*-infected ticks. In such epidemiological situations, babesiosis outbreaks can occur in adult cattle that were not naturally protected through parasite transmission by ticks before 10 months of age. In endemic areas, regular monitoring of the immune status of herds is recommended. If 75–80% of the 9–10-month-old cattle in a herd do not produce antibodies against *B. bovis*, vaccination is mandatory to prevent outbreaks [4]. Vaccination is also required when adult bovines are transported from tick-free to tick-endemic regions.

Currently available vaccines against bovine babesiosis are based on live attenuated parasites that are either amplified in vitro or in splenectomized calves. These vaccines are produced in several countries, including Argentina, Australia, Mexico, Brazil, Colombia, Israel and South Africa. They are recommended for use in bovines under a year of age and generally provide effective protection. However, if live vaccines are administered to adult animals, clinical symptoms of babesiosis can develop and lead to death if an infected animal is not treated [1, 2].

The production of live vaccines is cumbersome and time consuming and requires strict sanitary conditions to avoid the co-transmission of pathogenic microorganisms. Furthermore, vaccines must be kept chilled or frozen, which is particularly inconvenient in the tropical regions where they are often used. They also carry the risk of lack of protection against resistant strains, as was the case for Australian vaccines [5]. For these reasons, the development of a recombinant subunit vaccine would be a significant contribution to the control of bovine babesiosis. This type of vaccine is safer and simpler to produce on a large scale and therefore reduces the cost of production. Additionally, its use would not be limited to young cattle; it could be safely extended for use in adult animals. Experimental studies focused on the development of vaccines for *B. bovis* on the basis of recombinant antigens are scarce and have been limited to the use of prokaryotic expression systems. In some cases, they have been unsuccessful at using a single antigen [6, 7], whereas a formulation containing two recombinant sub-dominant antigens yielded promising results and provided a significant reduction in the parasitemia of challenged animals [8]. However, these animals developed clinical signs and had to receive treatment with parasiticide drugs. Likewise, immunization of cattle with a mixture of three recombinant merozoite surface antigens was not successful at preventing clinical signs of the disease after challenge [9].

Antigens anchored by glycosylphosphatidyl inositol (GPI) are candidates for vaccine development. In earlier studies, Florin-Christensen et al. [10] and Suarez et al. [11] described that *B. bovis* has at least five GPI-anchored antigens, belonging to the family of Merozoite Surface Antigen (MSA), which carry B-cell epitopes that are conserved between strains. Specific antibodies to these proteins prevent the invasion of erythrocytes and as such they play a vital role in parasite propagation [11–14]. Additionally, inhibition of GPI synthesis prevents in vitro growth of parasites in erythrocytes, corroborating the importance of these antigens in the process of invasion [15]. Furthermore, the release of GPI-anchored proteins from parasite cell membranes via treatment with phosphatidylinositol-specific phospholipase C prevents erythrocyte invasion by *B. bovis* merozoites [16]. As invasion of red blood cells is crucial to the survival of the parasite, blocking the antigens involved in this process may lead to protection. MSA-2c is highly conserved among *B. bovis* geographical isolates and therefore, together with its high immunogenicity during infection of cattle, it has been applied to the development of serological diagnostic methods [11, 13, 14, 17, 18].

Prokaryotic systems are widely used for recombinant protein production. However, heterologous eukaryotic proteins are not correctly modified, hampering their secretion significantly. Moreover, this is often accompanied by protein misfolding and segregation into insoluble inclusion bodies. Additionally, conformational epitopes, which may be critical for the production of protective antibodies, are likely to be absent in these proteins. Accordingly, the expression of recombinant proteins using eukaryotic systems may represent a long-term advantage in the effort to solve these problems.

A heterologous expression system in *Pichia pastoris* yeast has been used successfully in recent years. One of the advantages of this system is the ability to strictly regulate the expression of heterologous proteins and their secretion to the extracellular medium, facilitating their subsequent purification [19]. Additionally, in many cases, proteins retain their biological activity, making this approach useful for vaccine development [20, 21].

To generate a novel vaccine formulation against babesiosis, the ectodomains of MSA-2a_1_, MSA-2b and MSA-2c were produced as soluble recombinant proteins in *Pichia pastoris*. To test the potential of these vaccine candidates, immunization of mice with formulations of individual recombinant antigens that included the adjuvant Montanide ISA 720, and formulations comprising all three antigens and the adjuvant, were performed. Humoral and T-cell mediated immune responses were studied, and the ability of the immune sera to inhibit merozoite invasion in erythrocytes was evaluated.

## Methods

### Synthesis, cloning, and yeast expression

Synthetic genes encoding ectodomain amino acid residues 22–284 of MSA-2a_1_, residues 22–260 of MSA-2b and residues 25–239 of MSA-2c were synthetized by GenScript USA, Inc. (Piscataway, NJ, USA), with codon optimization to improve expression in *P. pastoris*. The amino acid sequences were based on MSA-2a_1_ (GenBank AAL15425), MSA-2b (GenBank AAL15427) and MSA-2c (GenBank AAL15428) of *B. bovis*. To prevent unwanted glycosylation, potential N-glycosylation sites were altered by using substitute amino acids. Constructs were designed to include appropriate restriction sites and a carboxyl-terminal hexa-histidine tag (His_6_) to enable purification. The synthetic genes that were cloned into the pUC57 vector were removed by digestion with an NotI enzyme mix (New England Biolabs, Ipswich, MA, USA) and subcloned into the NotI site of the *P. pastoris* expression vector pPIC9K (Invitrogen, Carlsbad, CA, USA). This expression vector contains a nucleotide sequence encoding the α-factor signal peptide of *Saccharomyces cerevisiae* to enable protein secretion, the AOX1 promoter for the control of gene expression, and the HIS4 gene for selection of recombinant yeast clones. Clones were selected that contained each gene in the correct orientation. Plasmids were linearized with SalI and were transformed into the GS115 strain (his4^−^) of *P. pastoris* by electroporation. Clones that were transformed with either plasmid pPIC9K-*BbMsa2a*_*1*_, pPIC9K-*BbMsa2b* or pPIC9K-*BbMsa2c* were screened for high copy-number integration by G418 selection; of these clones, two were resistant to 2–4 mg/ml of G418. Based on an immunoblotting analysis using an anti-His_6_ mAb, clones that were secreting high levels of each recombinant protein and which possessed a Mut^+^ phenotype were selected.

The expression and purification of the recombinant proteins was performed as described with several modifications [22]. A Mut^+^ transformant was initially grown overnight in 2 l of BMGY medium (1% w/v yeast extract, 2% w/v peptone, 1.34% w/v yeast nitrogen base without amino acids, 4 × 10^-5^% w/v biotin, 1% w/v glycerol, and 0.1 M potassium phosphate, pH 6.0) at 28–30 °C with vigorous shaking. The cells were harvested, resuspended in 400 ml BMMY (BMGY with glycerol replaced by 0.5% v/v methanol) and incubated again for 72 h. Methanol was added to a final concentration of 1% v/v every 24 h. After induction for 72 h, the cells were removed by centrifugation, and the culture supernatant was concentrated by ultrafiltration with an Amicon Ultracel 30,000 MWCO membrane (Millipore, Massachussets, USA) and extensively dialyzed at 4 °C against 20 mM sodium phosphate buffer/0.2 M NaCl, pH 8.0. The supernatant was applied to a column HisTrap FF coupled to a FPLC ÄKTA prime plus (GE Healthcare, Chicago, USA), which was previously equilibrated with 20 mM sodium phosphate/0.5 M NaCl, pH 8.0. Bound proteins were eluted with a 0 to 500 mM imidazole (Sigma-Aldrich, St. Louis, MO, USA) gradient in wash buffer (20 mM sodium phosphate/0.5 M NaCl/1 mM PMSF/10% glycerin, pH 8.0). Fractions containing protein were detected by SDS-PAGE and Coomassie blue staining, pooled, and used in a second-purification step by anionic exchange chromatography using Q FF resin coupled to a FPLC ÄKTA prime plus (GE Healthcare, Chicago, USA). The protein was eluted using a 0 to 1 M NaCl linear gradient and analyzed by SDS-PAGE. The peaks corresponding to each recombinant protein that had a high degree of purity were collected and dialyzed against phosphate-buffered saline (PBS). The protein concentration was determined by the Bradford method (BioRad, Richmond, CA, USA) using bovine serum albumin (BSA, Sigma-Aldrich, St. Louis, MO, USA) as a standard.

Purified proteins were analyzed by MALDI-TOF MS using a Microflex LT system (Bruker Daltonics, Bremen, Germany) and by reverse-phase high-performance liquid chromatography (RP-HPLC) using a Vydac C18 column (4.6 ×  250 mm; 300 μm particle size) coupled to an HPLC LCMS-2020 LC/MS system (Shimadzu, Kyoto, Japan). The HPLC procedure was performed at room temperature (RT) (*c*.25 °C) using a binary gradient of 0.1% trifluoroacetic acid (TFA, Solvent A) and 0.1% TFA in a 9:1 (v/v) solution of acetonitrile:water (Solvent B) with a two-step solvent gradient starting at 0 to 20% and followed by 20 to 100%, at a rate of 1 ml/min for 40 min. The elution was monitored with a UV-Visible absorbance detector (Shimadzu SPD M20A) at 220 and 280 nm.

### Circular dichroism

Circular dichroism (CD) was performed using a JASCO-J815 spectropolarimeter (Jasco, Tokyo, Japan) at 25 °C. Recombinant proteins were diluted to a concentration of 10 μM in PBS and loaded into a 5-mm quartz cuvette. Far-UV measurements (4 scans) were performed over wavelengths of 250–190 nm at intervals of 0.1 nm with a 1 nm bandwidth and 1 s response time. The spectra presented are the average of four scans, and the data obtained were reported as values of molar ellipticity [Ɵ]_MRW_ (deg × cm^2^ × dmol^-1^). A baseline measurement with buffer was subtracted from each spectrum. The secondary structure was estimated by computer analysis using the method of Raussens et al. [23] (http://perry.freeshell.org/raussens.html).

### Mice and immunization protocol

Female C57BL/6 mice at 6- to 8-weeks old were purchased from CEDEME (Federal University of São Paulo, Brazil) and used in all of the experiments. The immunogenicity of the recombinant proteins was evaluated in mice using homologous prime-boost protocols (protein prime/protein boost). Immunizations with the recombinant proteins were performed via the subcutaneous (s.c.) route with 1 or 10 μg of each protein emulsified in the presence of the adjuvant Montanide ISA 720 (Seppic, Puteaux, France), using proportions of 30/70 (vol/vol) of protein/adjuvant. Three injections were performed at three weeks apart. A volume of 50 μl was injected into each footpad (first dose) and a final volume of 100 μl was injected at the base of the tail (second and third doses). After each immunization, blood was collected from the tail, and the sera were analyzed for the presence of antibodies against each recombinant protein.

### Immunological assays

#### Immunoblotting analysis

Proteins were fractionated by 12% SDS-PAGE under reducing conditions and were transferred from the gel to nitrocellulose membranes (Hybond N, GE Healthcare, Chicago, USA) with the aid of a Mini Trans-Blot apparatus (BioRad, Richmond, CA, USA). The immunoblot was essentially performed as described [24]. The membranes were incubated with mouse monoclonal anti-histidine tag antibodies (anti-His tag, GE Healthcare, Chicago, USA) at a final dilution of 1:1,000. After 1 h at RT, membranes were washed, and goat anti-mouse Immunoglobulin G (IgG) coupled to peroxidase was added to the membranes at a final dilution of 1:2,000 (Sigma-Aldrich, St. Louis, MO, USA). After 1 h incubation at RT, the reaction was developed using a chemiluminescence detection assay (ECL, GE Healthcare, Chicago, USA).

#### Determination of mouse antibody titers against *B. bovis* recombinant protein

Antibodies against each recombinant protein in mouse sera were detected by enzyme-linked immunosorbent assay (ELISA), essentially as described [24]. The recombinant proteins were employed as solid phase-bound antigens (200 ng/well), and a volume of 50 μl of each solution was added to each well of a 96-well plate. After overnight incubation at RT, plates were washed with a solution of PBS and 0.05% Tween-20 (PBS-T) and blocked with a solution of PBS, 2.5% (w/v) skimmed milk and 2.5% BSA for 2 h at 37 °C. Serial dilutions of murine polyclonal sera (100 μl) were added to wells in duplicates, followed by incubation for 1 h at RT. After washing with PBS-T, 50 μl aliquots of peroxidase-labeled goat anti-mouse IgG (Sigma-Aldrich, St. Louis, MO, USA), diluted 1:1,000, were added to each well. Detection by chemiluminescence was carried out as above. Anti-MSA titers were determined based on the highest dilution of sera that yielded an A_492_ higher than 0.1. For detection of IgG subclass responses, secondary antibodies specific to mouse IgG1, IgG2b, and IgG2c were used (Southern Technologies, Chattanooga, Tennessee, USA). Results are expressed as the mean values of IgG titers (log_10_) ± standard error (SEM) that were detected 2 weeks after each immunizing dose.

#### Indirect immunofluorescence assay

Immunofluorescence assays were performed as described [18]. Briefly, smears were prepared using a suspension of *B. bovis* (BboS2P pathogenic strain, Argentina)-parasitized erythrocytes (13% infection) that were cultured in vitro and diluted 1:3 in PBS containing 1% BSA. The preparations were fixed with cold methanol for 15 min and blocked with 3% BSA in PBS for 30 min at 37.5 °C in a humidified incubator. Sera from mice immunized with the recombinant proteins (dilution 1:50) were applied to the slides and incubated for 1 h. Slides were then washed 3 times with PBS before the addition of either anti-mouse IgG conjugated to fluorescein isothiocyanate (FITC) (Sigma-Aldrich, St. Louis, MO, USA), which was diluted 1:500 in PBS containing 3% BSA, or 4’,6-Diamidino-2-Phenylindole Dihydrochloride (DAPI, Invitrogen, Carlsbad, CA, USA). Positive anti-rMSA-2c (13) and negative (non immunized mice) reference sera were included on each slide. Binding was visualized using a Nikon TS 100 epifluorescence microscope.

#### Invasion inhibition assay

*Babesia bovis* merozoites of the Argentine S2P pathogenic strain (BboS2P) were cultured in vitro on bovine erythrocytes, as described previously [16]. Growth was monitored by microscopic observation of Giemsa-stained smears. Inhibition of merozoite invasion of erythrocytes was carried out as described for the strain R1A [13] with some modifications. Briefly, aliquots of BboS2P-infected bovine erythrocyte cultures were incubated in triplicate in 96-well plates either with pools of control mouse sera (from five mice, ELISA titer < 10^2^) or with immune sera (from five mice each, ELISA titers > 10^4^), diluted 1:10 in culture medium, at 37 °C in a 5% CO_2_ atmosphere. The packed volume of bovine erythrocytes was 5% (v/v), and the initial percentage of parasitized erythrocytes (PPE) was approximately 1%. All sera had been heat-inactivated for 30 min at 56 °C. At 24 h and 48 h, supernatants (80% of the total volume of each well) were replaced with fresh culture medium containing the corresponding murine sera. Percentages of parasitized erythrocytes were determined at 72 h by microscopic examination of 2500 erythrocytes in Giemsa-stained smears that were prepared from each well.

#### Cytokine measurement

To determine the intracellular expression of cytokines interferon gamma (IFN-γ) and tumor necrosis factor (TNF-α), intracellular cytokine staining (ICS) was used. Splenocytes and lymph node cells (popliteal and inguinal) that were collected from C57BL/6 mice 12 days after the third dose were treated with ammonium-chloride-potassium buffer. ICS was evaluated following the in vitro culture of splenocytes in the presence or absence of antigenic stimulus. Cells were washed 3 times in RPMI 1640 medium (pH 7.4) and re-suspended in cell culture medium consisting of RPMI 1640 medium (pH 7.4) that was supplemented with 10 mM HEPES, 0.2% sodium bicarbonate, 59 mg/l of penicillin, 133 mg/l of streptomycin, and 10% fetal bovine serum (Hyclone, Logan, USA). Cell viability was evaluated using 0.2% trypan blue exclusion dye. Cell density was adjusted to 5 × 10^6^ cells/ml in cell culture medium containing anti-CD28 (2 μg/ml), BDGolgiPlug (10 μg/ml) and monensin (5 μg/ml). In half of the cultures, either a final concentration of 10 μg/ml of the indicated recombinant proteins or 2 μg/ml of Concanvalin A (ConA, Sigma-Aldrich, St. Louis, MO, USA) were added. The cells were cultivated in V-bottom 96-well plates (Corning, Tewksbury, MA, USA) in a final volume of 200 μl in duplicate, at 37 °C in a humid environment containing 5% CO_2_. After a 12 h incubation, cells were stained for surface markers with PerCP-Cy5.5-labeled anti-CD4 (clone RM4-5), or PECy7-labeled CD8 (clone 53–6.7), on ice for 20 min. To detect IFN-γ and TNF-α by intracellular staining, cells were then washed twice in PBS/0.5% BSA/2 mM EDTA, fixed and permeabilized with BD perm/wash buffer (BD Biosciences, San Jose, CA, USA). After being washed twice with BD perm/wash buffer, cells were stained for intracellular markers using APC-labeled anti-IFN-γ (Clone XMG1.2) and PE-labeled anti-TNF-α (clone MP6-XT22). Finally, cells were washed twice with BD perm/wash buffer and fixed in 1% PBS-paraformaldehyde. At least 300,000 cells were acquired on a BD FACSCanto II flow cytometer and then analyzed with FlowJo 8.7 software (http://www.flowjo.com/).

To measure the number of cells secreting IFN-γ, splenocytes and lymph node cells collected from immunized C57BL/6 mice were used for enzyme-linked immunospot (ELISPOT) assays. These assays were performed as in previous studies [25].

### Statistical analysis

Values were log transformed and compared using One-Way ANOVA followed by Tukey’s HSD tests (http://faculty.vassar.edu/lowry/VassarStats.html). Differences were considered significant when *P*-values were < 0.05.

## Results

### Expression, purification, and biochemical characterization of rMSA-2a_1_, rMSA-2b, and rMSA-2c

**Fig. 1:**
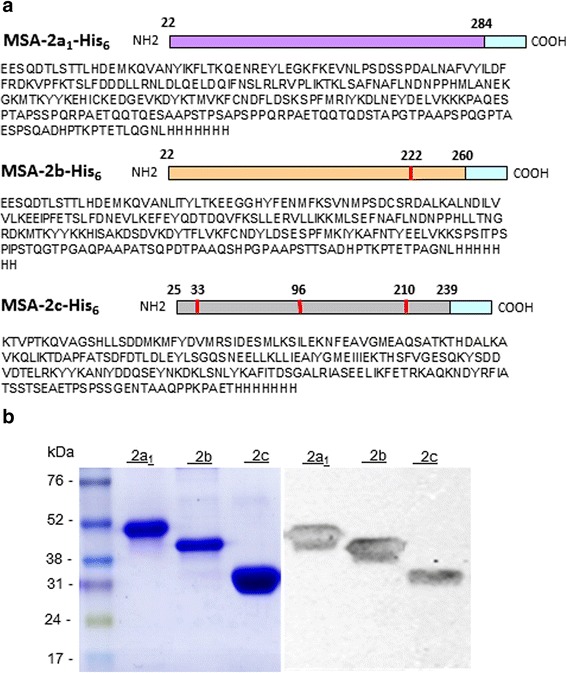
Expression of *B. bovis* merozoite surface antigens as soluble recombinant proteins in *P. pastoris*. **a** Schematic representation of MSA-2a_1_-His_6_, MSA-2b-His_6_ and MSA-2c-His_6_. Codon-optimized genes were synthesized and contained nucleotides corresponding to the indicated primary protein structures. Amino acids indicated in *red* represent modifications that were made to avoid N-glycosylation. **b** Genes were expressed as fusion recombinant proteins containing a hexa-histidine tag (His_6_) in *Pichia pastoris* yeast. The supernatants of yeast clones expressing each recombinant merozoite antigen were separated by SDS-PAGE under reducing conditions, stained with Coomassie Blue (*left panel*) and immunoblotted on nitrocellulose membranes using antibodies against the His_6_ tag (*right panel*). Lanes 1 and 4: MSA-2a_1_-His_6_; Lanes 2 and 5: MSA-2b-His_6_; Lanes 3 and 6: MSA-2c-His_6_

For secreted expression in the methylotrophic yeast *Pichia pastoris*, constructs based on the codon-optimized gene sequences of MSA-2a_1_, MSA-2b and MSA-2c were used. The amino acid sequences of the expressed ectodomains of each protein are shown in Fig. 1a. MSA-2b and MSA-2c amino acid sequences have one and three putative N-linked glycosylation (NxS/T) sites, respectively, and substitute amino acids (NxS/T to AxS/T) were introduced to remove these sites. The final constructs also encoded an N-terminal hexa-histidine tag for Ni^2+^-chelating chromatography. The synthetic *msa-2a*_*1*_, *msa-2b* and *msa-2c* genes were sub-cloned in frame with the yeast α-factor secretion signal peptide into the expression vector pPIC9K.

The generated recombinant proteins were soluble and secreted into the extracellular medium. Figure 1b shows SDS-PAGE analyses of supernatants originated from yeast clones for each recombinant protein (left panel). The presence of recombinant proteins was demonstrated by immunoblotting using anti-His_6_-mAb (right panel).

**Fig. 2:**
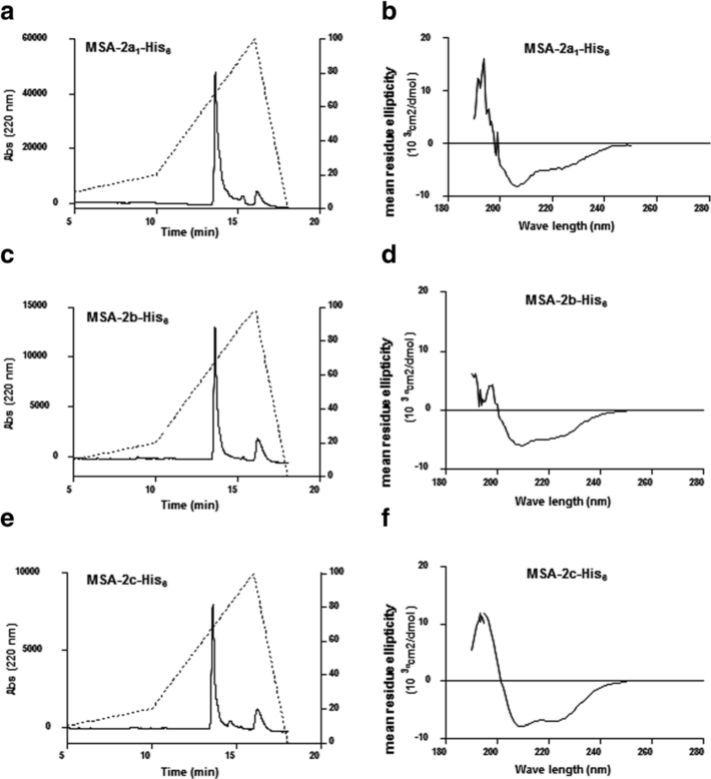
Biochemical characterization of recombinant *B. bovis* merozoite antigens. rMSA-2a_1_, rMSA-2b and rMSA-2c were analyzed by RP-HPLC (**a**, **c** and **e**). A main peak was detected in each case, denoting a high level of purity. The circular dichroism spectrum of each recombinant protein was recorded from 190 to 250 nm using a JASCO-J815 spectropolarimeter (**b**, **d** and **f**). The plots represent the mean residue ellipticity of the recombinant proteins

After purification, yields of up to 20 mg/l of culture were obtained for rMSA-2a_1_, rMSA-2b and rMSA-2c. A high protein purity was confirmed by reverse-phase chromatography on a C-18 column by observation of single peaks, as shown in Fig. 2a, c, e.

To investigate the secondary structures of the recombinant products, far-UV CD spectroscopy was performed (Fig. 2b, d, f). In all cases, CD spectra were consistent with folded proteins. The following estimated percentages of α-helix, anti-parallel β-sheet, parallel β-sheet and β-turn for each protein were found: (i) MSA-2a_1_-His_6_: 46% α-helix, 19% β-sheet, 12.5% β-turn; (ii) MSA-2b-His_6_: 8.9% α-helix, 24.4% β-sheet, 12.5% β-turn; (iii) MSA-2c-His_6_: 34% α-helix, 13% β-sheet, 12.5% β-turn.

### Immunogenic properties of rMSA-2a_1_, rMSA-2b and rMSA-2c in mice

**Fig. 3:**
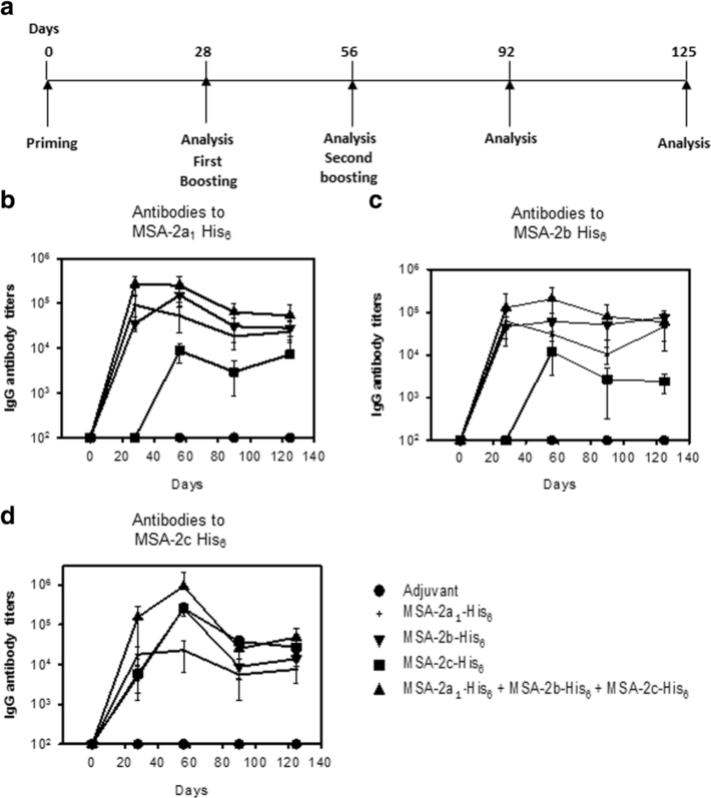
Specific antibody responses in mice immunized with recombinant *B. bovis* merozoite antigens. Immunization of C57BL/6 mice and measurement of antibody titers by ELISA was carried out according to the timeline described in **a**. Mice were divided in five groups (*n* = 5 in each group) and immunized with three doses of the following: (1) Montanide ISA 720; (2) Montanide ISA 720 plus rMSA-2a_1_ (10 μg/dose/mouse); (3) Montanide ISA 720 plus rMSA-2b (10 μg/dose/mouse); (4) Montanide ISA 720 plus rMSA-2c (10 μg/dose/mouse); (5) Montanide ISA 720 plus a protein mix (rMSA-2a_1_, rMSA-2b, rMSA-2c; 10 μg of each protein per mouse). Serum IgG antibody titers against *B. bovis* merozoite antigens were determined by ELISA using each recombinant protein in the plate. **b** rMSA-2a_1._ **c** rMSA-2b. **d** rMSA-2c. The results represent the mean values ± standard error obtained for each group

Initially, mice were immunized either with each individual recombinant protein or with all three recombinant proteins combined using a 1 μg dose per mouse. However, the antibody titers did not reach desirable levels to all antigens (data not shown). Thus, in order to increase immunogenicity, we decided to immunize mice with 10 μg of each recombinant protein per mouse per dose or a combination of all three recombinant proteins, according to the schedule described in Fig. 3a. The comparative immunogenicities of each animal group (*n* = 5) were determined by the mean values + SE of the serum IgG antibody titers against each of the recombinant proteins, as estimated by ELISA.

Antibody titers of up to ~ 10^5^ against the homologous protein were observed in mice immunized with rMSA-2a_1_ (Fig. 3b). The antibody titers against the other recombinant proteins were also high, in the range of ~ 10^5^ or ~ 10^4^ for rMSA-2b or rMSA-2c, respectively (Fig. 3c, d).

The immunization of mice with rMSA-2b elicited high antibody titers to all three recombinant proteins (~ 10^5^, Fig. 3b-d). Likewise, mice immunized with rMSA-2c displayed significant antibody titers to all three recombinant proteins (~ 10^5^, Fig. 3b-d). Most relevant for vaccine development was the fact that mice immunized with the three recombinant proteins admixed responded extremely well to all three recombinant proteins, with antibody titers that were higher than 10^5^ with respect to both rMSA-2a_1_ and rMSA-2b (Fig. 3b, c) and even higher with respect to rMSA-2c (~10^6^, Fig. 3d). Their antibody titers were the highest observed out of all of the groups of immunized mice to each of the recombinant proteins, demonstrating that there was an additive effect (Fig. 3b-d). These antibody titers were long-lasting and could be detected until day 125 after the first dose. As in the previous experiment, control mice injected with adjuvant alone did not present specific detectable antibodies (Fig. 3b-d).

**Fig. 4:**
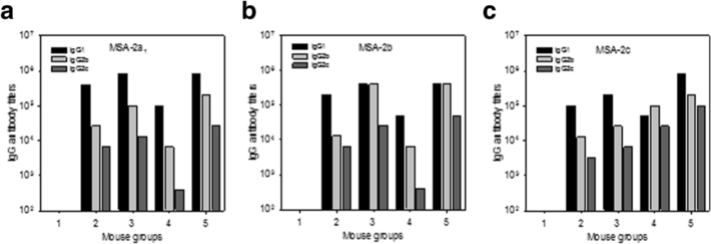
IgG sub-classes of specific antibodies in mice immunized with recombinant *B. bovis* merozoite antigens. Mice were immunized as described in Fig. 3. Antibody titers of each IgG subclass (IgG1, IgG2b, IgG2c) to each *B. bovis* merozoite antigen were determined by using a pool of sera from each mouse group. **a** rMSA-2a_1._ **b** rMSA-2b. **c** rMSA-2c. These data represent one of two independent experiments

IgG sub-classes were also estimated. Except for Gr. 1 (adjuvant only), all other mouse groups developed high IgG1 antibody titers to all three recombinant proteins (Fig. 4a-c).

In conclusion, the three *B. bovis* recombinant proteins adjuvanted in Montanide ISA 720 were highly immunogenic in mice.

### *Babesia bovis* merozoite recognition and ability to inhibit parasite invasion with rMSA-2a1, rMSA-2b and rMSA-2c immune sera

**Fig. 5:**
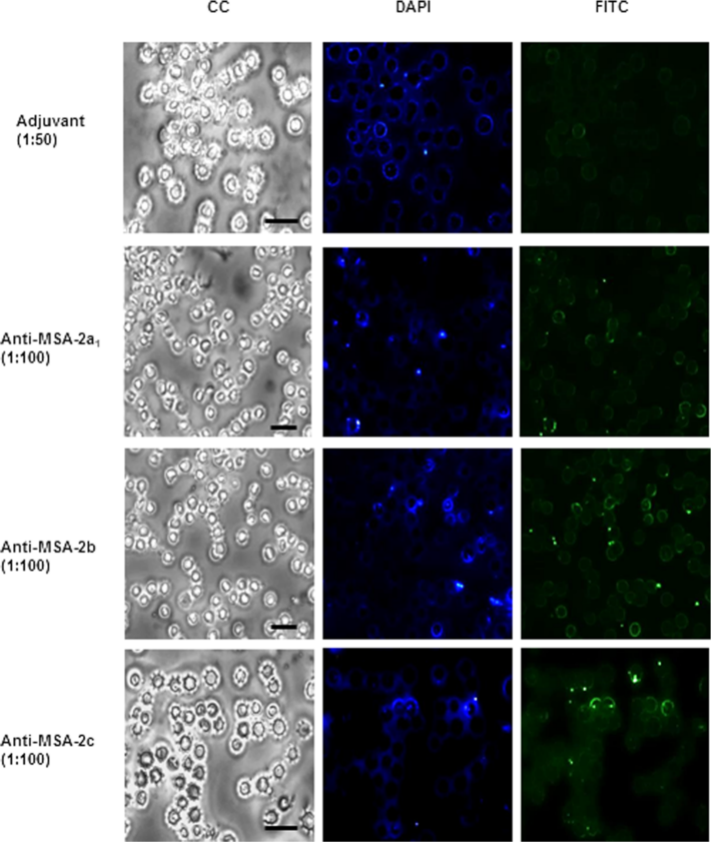
Indirect immunofluorescence assay for the recognition of native MSA-2a_1_, MSA-2b and MSA-2c. Smears of *B. bovis*-infected erythrocytes (CC) were incubated with pools of sera from mice immunized with each recombinant protein (1:100 dilutions) or control sera (1:50 dilution). Reactions were detected with FITC-conjugated anti-mouse IgG antibodies (FITC). Nuclei were detected by DAPI staining (DAPI). Slides were observed with 400× magnification. *Scale-bars*: 10 μm

As a next step, we investigated if antibodies against the three recombinant proteins reacted with *B. bovis* merozoites using immunofluorescence assays. We observed that the sera from each of the groups of mice immunized with the recombinant proteins reacted with merozoites of *B. bovis* at 1:100 dilutions (Fig. 5b-d). Antibody recognition was specific, as control sera from mice immunized with adjuvant only were not reactive, even at a 1:50 dilution (Fig. 5a).

**Fig. 6:**
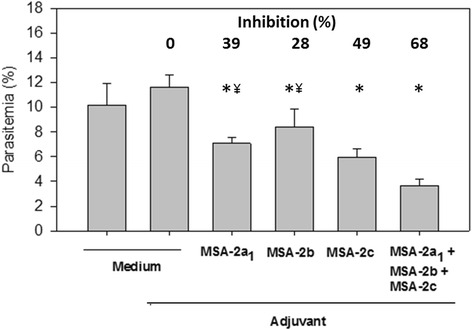
Inhibition of erythrocyte invasion by sera from mice immunized with the different antigen formulations. Mice were immunized as described in Fig. 3. Pooled sera of the different mice groups were added (1:10, final concentration) in triplicate to wells containing *B. bovis*-infected erythrocytes that were cultured in vitro. Percentages of parasitized erythrocytes (PPE) were evaluated in Giemsa-stained smears. The results are expressed as the mean PPE ± standard error values that were obtained after 72 h in each condition. The results are representative of two independent experiments. Asterisks denote statistically significant differences in PPE: * *P* < 0.001 for rMSA-2a_1_ sera; *P* = 0.008 for rMSA-2b sera; *P* < 0.001 for rMSA-2c sera; *P* < 0.001 for the sera from mixture of three proteins; compared to cultures incubated with medium only; and ^¥^ *P* = 0.006 compared to cultures incubated with sera from mice immunized with the mixture of three proteins

We subsequently evaluated the immune sera for its ability to inhibit merozoite invasion of bovine erythrocytes in culture. The presence of immune sera collected from mice immunized with rMSA-2a_1_, rMSA-2b or rMSA-2c led to significative specific inhibitions of 39, 28 and 49% of erythrocyte invasion, respectively (Fig. 6, ANOVA: *F*_(4,10)_ = 33.66, *P* <0.001 for rMSA-2a_1_ and rMSA-2c; *P* = 0.008 for rMSA-2b). However, most relevant for vaccine development was the fact that sera obtained from mice vaccinated with all three recombinant proteins admixed inhibited parasite invasion by 68% (Fig. 6, ANOVA: *F*_(4,10)_ = 33.66, *P* < 0.001). This inhibition was specific because sera from mice injected with adjuvant only failed to inhibit parasite invasion of erythrocytes.

### Cell-mediated immune responses to rMSA-2a_1_, rMSA-2b and rMSA-2c of *B. bovis*

**Fig. 7:**
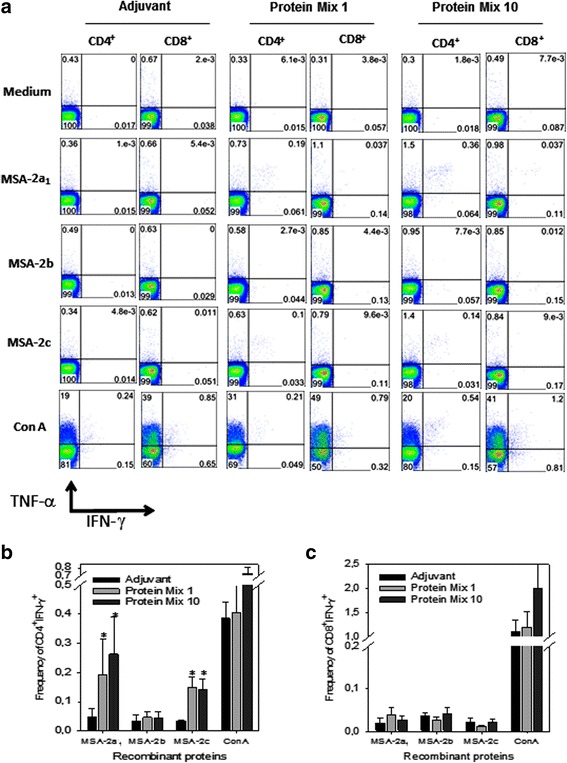
Cell-mediated immunity of mice immunized with recombinant MSA antigens. C57BL/6 mice were immunized with three doses, 21 days apart, of a mixture containing either 1 μg or 10 μg of each protein/dose (per mouse) emulsified in Montanide ISA 720. Control mice received adjuvant only. Splenic cells of 4 mice were cultured in the presence of anti-CD28, monensin and brefeldin-A with or without the recombinant proteins or ConA, as indicated. After 12 h, cells were stained with anti-CD4, anti-CD8, anti-IFN-γ, and anti-TNFα. **a** Total frequencies (%) of CD4^+^ or CD8^+^ cells stained for TNF-α and IFN-γ **b** and **c** Mean frequencies of 4 mice ± SE of IFN-γ^+^ CD4 or CD8 cells, respectively. The results are representative of two experiments. *Asterisks* denote statistically significant (*P* = 0.009 for rMSA-2a_1_ and *P* = 0.045 for rMSA-2c) higher frequencies of cells from mice immunized with the mixture of proteins when compared to cells from mice injected with adjuvant only

To determine whether immunization with the recombinant proteins elicited T cell-mediated immune responses, spleen and lymph node cells were collected from 4 mice immunized either with mixture of three proteins emulsified with adjuvant or with adjuvant alone. Cells were stimulated ex vivo with recombinant proteins, after which IFN-γ and TNF-α expression was measured by intra-cellular staining (ICS) and the number of IFN-γ-secreting cells was assessed by ELISPOT. The results showed that splenic cells of mice immunized with the mixture of three proteins (either 1 μg or 10 μg of each protein per dose per mouse) and re-stimulated in vitro with rMSA-2a_1_ or rMSA-2c had a significant increase in the frequencies of CD4^+^ T cells expressing IFN-γ and IFN-γ/TNF-α (Fig. 7a, b; ANOVA: *F*_(3,12)_ = 6.89, *P* = 0.009 for CD4^+^ IFN-γ^+^ cells and *F*_(3,12)_ = 9.25, *P* = 0.015 for CD4^+^ IFN-γ^+^/TNF-α^+^ cells). The same significant increase in the frequencies of CD4^+^ T cells was observed when splenic cells were re-stimulated with rMSA-2c (Fig. 7a, b; ANOVA: *F*_(3,12)_ = 6.89, *P* = 0.045 for CD4^+^ IFN-γ^+^ cells and *F*_(3,12)_ = 9.25, *P* = 0.019 for CD4^+^ IFN-γ^+^/TNF-α^+^ cells). In contrast, we could not detect a significant increase in the IFN-γ^+^ or IFN-γ^+^TNF-α^+^ cells upon re-stimulation with rMSA-2b in vitro or in CD8^+^ T cells (Fig. 7a-c; ANOVA: *F*_(3,12)_ = 6.89, *P* = 0.874 for CD4^+^ IFN-γ^+^ cells and *F*_(3,12)_ = 9.25, *P* = 0.383 for CD4^+^ IFN-γ^+^/TNF-α^+^ cells).

Similar results were observed when lymph node cells were re-stimulated with the recombinant proteins. Cells of mice immunized with mixture of proteins (either 1 μg or 10 μg of each protein per dose per mouse) and re-stimulated in vitro with rMSA-2a_1_ or rMSA-2c had a significant increase in the frequencies of CD4^+^ T cells expressing IFN-γ and IFN-γ/TNF-α (ANOVA: *F*_(3,12)_ = 29.11, *P* = 0.021 for rMSA-2a_1_ and *P* < 0.001 for rMSA-2c) (Additional file 1: Figure S1a).

In parallel, these cells were assayed via ELISPOT. Similar to the ICS experiments, significant differences in the frequencies of IFN-γ-producing cells were detected when we analyzed the splenic or lymph node cells (Additional file 1: Figure S1b, c respectively) of mice immunized with the mixture of proteins (either 1 μg or 10 μg per dose per mouse).  A significantly higher number of cells from mice immunized with the protein mix secreted IFN-γ upon in vitro re-stimulation with rMSA-2a_1_ (ANOVA: *F*_(3,12)_ = 29.23, *P* = 0.002 for splenic cells and *F*_(3, 12)_ = 43.53, *P* < 0.001 for lymph node cells) or rMSA-2c (ANOVA: *F*_(3,12)_ = 29.23, *P* < 0.001 for splenic cells and *F*_(3,12)_ = 43.53, *P* < 0.001 for lymph node cells). In contrast, these cells failed to secrete this cytokine upon stimulation with rMSA-2b (ANOVA: *F*_(3,12)_ = 29.23, *P* = 0.989 for splenic cells and *F*_(3,12)_ = 43.53, *P* = 0.251 for lymph node cells). We concluded that, following protein immunization, T cell-mediated immunity to the recombinant proteins MSA-2a_1_ or MSA-2c of *B. bovis* can be detected in the spleens and lymph nodes of the immunized mice.

## Discussion

Our study describes a new multi-epitope vaccine formulation against *B. bovis* based on three soluble recombinant proteins produced in *P. pastoris* yeast. This platform optimized protein expression, facilitated purification and yielded high quantities of folded recombinant proteins at a high degree of purity. The predominance of α-helix in MSA-2a_1_ and MSA-2c may be essential to their functioning and is compatible with the predicted structures of the native proteins [14]. This fact could be important for vaccine development as thus far recombinant proteins produced in bacteria have failed to provide protection upon challenge when used as vaccines in cattle [2]. The recombinant MSA-2b protein showed a higher degree of a random secondary structure than the others. Accordingly, this protein was less immunogenic than rMSA-2a_1_ and rMSA-2c, both in B-cell and T-cell mediated responses. These results support the idea of a close relationship between protein folding and protein function. The successful use of the *P. pastoris* expression system may be significant for vaccine development efforts against *B. bovis* because there are other molecules, such as apical membrane antigen-1 or rhoptry-associated protein-1, that could also be expressed and purified applying this strategy [26–28].

For our multi-epitope vaccine formulation, we selected three MSAs. These antigens are GPI-anchored membrane proteins and carry B-cell epitopes, which are conserved between strains, and they target specific antibodies that prevent the invasion of erythrocytes [11–14]. Interestingly, our work clearly shows immunological cross-reactivity between the three MSAs tested, which can be partially explained on the basis of amino acid similarities. Although all N-glycosylation sites have been removed in the recombinant proteins, we cannot exclude the possibility of these proteins sharing post-translational modifications as source of antigenic similarities. However, immunological cross-reactivity was not observed when these proteins were expressed in a prokaryotic system, where proteins are mostly expressed misfolded [10]. Thus, we suggest that this observation is likely due to the presence of B-cell epitopes that are generated in the *P. pastoris* system [14] and would be absent in a prokaryotic system.

Supporting these earlier studies, we observed that immune sera obtained from mice immunized with our vaccine formulation inhibited the growth of parasites in vitro in bovine erythrocytes. Although it is so far unknown the specific role of MSAs proteins during parasite invasion, the neutralization of some surface proteins through antibodies could interfere with the invasion process of erythrocytes. As this is an important step for parasite survival, these antibodies may be capable to control parasitemia in vivo, which in turn may alleviate disease symptoms. Our results show that MSA-specific immune sera are able to partially neutralize parasite invasion in vitro, suggesting a potential participation of those proteins during cell invasion. Importantly, the immunization with a mixture of all three MSAs improved the neutralization activity of immune sera, probably due to a broader breadth of the recognized parasitic epitopes.

In addition to invasion-inhibitory antibodies, the importance of Th1 cells in the development of immunity against *B. bovis* has been proposed. In cattle infected with *B. bovis* and *B. bigemina*, the main cellular response is Th1 type, and it was hypothesized that such response could induce protection against challenge. In this case, the Th1 response would be mediated by effector CD4^+^ T cells that secrete IFN-γ and provide help for the production of protective antibodies [3, 29]. In this study, the frequencies of IFN-γ and IFN-γ/TNF-α-expressing cells were quantified. Our vaccine formulation elicited easily detectable frequencies of CD4^+^ T cells expressing IFN-γ and IFN-γ/TNF-α in the spleens and lymph nodes of immunized mice. In the case of MSA-2b, the ratio IgG1/IgG2 = 1 found in mice immunized with this recombinant protein should indicate a balanced Th1/Th2 response. We were not able to detect cellular responses elicited by this antigen; however, we believe that it is worth to maintain MSA-2b in the vaccine formulation, as a significative invasion inhibition by sera from mice immunized with MSA-2b only was observed.

Our results are encouraging to test this vaccine in cattle, as it remains to be evaluated whether this formulation which elicited such response in mice, will be able to elicit enough neutralizing MSA-specific antibodies and/or T cell-mediated responses to obtain a complete protection in cattle.

## Conclusion

In summary, we have shown that a multi-epitope vaccine is immunogenic to CD4^+^T cells and can generate high levels of antibodies and T cell-mediated immunity. This formulation will be used in future work to determine whether it provides protection against bovine babesiosis.

## Additional file

Additional file 1: Figure S1. Cell-mediated immunity of mice immunized with recombinant MSAs. C57BL/6 mice were immunized as described in Fig. 7. Splenic or lymph node cells of these mice were cultured in the presence or absence of the recombinant proteins or ConA, as indicated. **a** The lymph node cells of these mice were cultured in the presence of anti-CD28, monensin and brefeldin-A with or without the recombinant proteins or ConA, as indicated. After 12 h, cells were stained with anti-CD4 and anti-IFN-γ. The results are expressed as the mean ± SE of the total frequency (%) of CD4^+^ IFN-γ ^+^ cells from 4 mice. **b**, **c** After 48 h, IFN-γ secreting cells (spot forming cells, SFC) were estimated by ELISPOT assay. The results are representative of two experiments and are expressed as number of SFC per 5 × 10^5^ splenic (**b**) or lymph node (**c**) cells obtained from 4 mice. Asterisks denote statistically significant (**b**: *P* = 0.002 for rMSA-2a1; *P* < 0.001 for rMSA-2c; and **c**: *P* < 0.001 for rMSA-2a_1_; *P* < 0.001 for rMSA-2c) higher frequencies of number of cells from mice immunized with the mixture of proteins compared to cells from mice injected with adjuvant only. (TIF 49 kb)

## Abbreviations

BSA: Bovine serum albumin
CD: Circular dichroism
ConA: Concanvalin A
DAPI: 4’,6-diamidino-2-phenylindole dihydrochloride
ELISA: Enzyme-linked immunosorbent assay
ELISPOT: Enzyme-linked immunospot
FITC: Fluorescein isothiocyanate
FPLC: Fast protein liquid chromatography
GPI: Glycosylphosphatidyl inositol
His_6_: Hexa-histidine tag
ICS: Intracellular cytokine staining
IFN-γ: Interferon gamma
IgG: Immunoglobulin G
MSA: Merozoite surface antigen
PBS: Phosphate-buffered saline
PBS-T: Phosphate-buffered saline 0.05% Tween-20
PPE: Percentage of parasitized erythrocytes
RP-HPLC: Reverse-phase high-performance liquid chromatography
RT: Room temperature
SDS-PAGE: Sodium dodecyl sulfate polyacrylamide gel electrophoresis
TFA: Trifluoroacetic acid
TNF-α: Tumor necrosis factor alpha

## References

[1] Bock RE, de Vos AJ, Lew A, Kingston TG, Fraser IR (1995). Studies on failure of T strain live *Babesia bovis* vaccine. Aust Vet J.

[2] Florin-Christensen M, Suarez CE, Rodriguez AE, Flores DA, Schnittger L (2014). Vaccines against bovine babesiosis : where we are now and possible roads ahead. Parasitology.

[3] Brown WC, Norimine J, Knowles DP, Goff WL (2006). Immune control of *Babesia bovis* infection. Vet Parasitol.

[4] de Waal DT, Combrink MP (2006). Live vaccines against bovine babesiosis. Vet Parasitol.

[5] Bock R, Jackson L, deVos A, Jorgensen W (2004). Babesiosis of cattle. Parasitology.

[6] Hines SA, Palmer GH, Jasmer DP, Goff WL, McElwain TF (1995). Immunization of cattle with recombinant *Babesia bovis* merozoite surface antigen-1. Infect Immun.

[7] Norimine J, Mosqueda J, Suarez C, Palmer GH, McElwain TF, Mbassa G (2003). Stimulation of T-helper cell gamma interferon and immunoglobulin G responses specific for *Babesia bovis* rhoptry-associated protein 1 (RAP-1) or a RAP-1 protein lacking the carboxy-terminal repeat region is insufficient to provide protective immunity again. Infect Immun.

[8] Hope M, Riding G, Menzies M, Colditz I, Reverter A, Willadsen P (2005). Potential for recombinant *Babesia bovis* antigens to protect against a highly virulent isolate. Parasite Immunol.

[9] Alvarez JA, Lopez U, Rojas C, Borgonio VM, Sanchez V, Castañeda R, et al. Immunization of *Bos taurus* steers with *Babesia bovis* recombinant antigens MSA-1, MSA-2c and 12D3. Transbound Emerg Dis. 2010;57:87–90. 10.1111/j.1865-1682.2010.01117.x 20537116

[10] Florin-Christensen M, Suarez CE, Hines SA, Palmer GH, Brown WC, McElwain TF (2002). The *Babesia bovis* merozoite surface antigen 2 locus contains four tandemly arranged and expressed genes encoding immunologically distinct proteins. Infect Immun.

[11] Suarez CE, Florin-Christensen M, Hines SA, Palmer GH, Brown WC, McElwain TF (2000). Characterization of allelic variation in the *Babesia bovis* merozoite surface antigen 1 (MSA-1) locus and identification of a cross-reactive inhibition-sensitive MSA-1 epitope. Infect Immun.

[12] Mosqueda J, McElwain TF, Stiller D, Palmer GH (2002). *Babesia bovis* merozoite surface antigen 1 and rhoptry-associated protein 1 are expressed in sporozoites, and specific antibodies inhibit sporozoite attachment to erythrocytes. Infect Immun.

[13] Wilkowsky SE, Farber M, Echaide I, Torioni De Echaide S, Zamorano PI, Dominguez M (2003). *Babesia bovis* merozoite surface protein-2c (MSA-2c) contains highly immunogenic, conserved B-cell epitopes that elicit neutralization-sensitive antibodies in cattle. Mol Biochem Parasitol.

[14] Dominguez M, Echaide I, de Echaide ST, Mosqueda J, Cetrá B, Suarez CE (2010). In silico predicted conserved B-cell epitopes in the merozoite surface antigen-2 family of *B. bovis* are neutralization sensitive. Vet Parasitol.

[15] Rodríguez AE, Couto A, Echaide I, Schnittger L, Florin-Christensen M (2010). *Babesia bovis* contains an abundant parasite-specific protein-free glycerophosphatidylinositol and the genes predicted for its assembly. Vet Parasitol.

[16] Rodriguez AE, Florin-Christensen M, Flores DA, Echaide I, Suarez CE, Schnittger L (2014). The glycosylphosphatidylinositol-anchored protein repertoire of *Babesia bovis* and its significance for erythrocyte invasion. Ticks Tick Borne Dis.

[17] Kim C-M, Blanco LBC, Alhassan A, Iseki H, Yokoyama N, Xuan X (2008). Development of a rapid immunochromatographic test for simultaneous serodiagnosis of bovine babesioses caused by *Babesia bovis* and *Babesia bigemina*. Am J Trop Med Hyg.

[18] Dominguez M, Echaide I, de Echaide ST, Wilkowsky S, Zabal O, Mosqueda JJ (2012). Validation and field evaluation of a competitive enzyme-linked immunosorbent assay for diagnosis of *Babesia bovis* infections in Argentina. Clin Vaccine Immunol.

[19] Cereghino GPL, Cereghino JL, Ilgen C, Cregg JM (2002). Production of recombinant proteins in fermenter cultures of the yeast *Pichia pastoris*. Curr Opin Biotechnol.

[20] Macauley-Patrick S, Fazenda ML, McNeil B, Harvey LM (2005). Heterologous protein production using the *Pichia pastoris* expression system. Yeast.

[21] Balamurugan V, Reddy GR, Suryanarayana VVS (2007). *Pichia pastoris*: A notable heterologous expression system for the production of foreign proteins - Vaccines. Indian J Biotechnol.

[22] Vicentin EC, Françoso KS, Rocha MV, Iourtov D, Dos Santos FL, Kubrusly FS (2014). Invasion-inhibitory antibodies elicited by immunization with *Plasmodium vivax* apical membrane antigen-1 expressed in *Pichia pastoris* yeast. Infect Immun.

[23] Raussens V, Ruysschaert J-M, Goormaghtigh E (2003). Protein concentration is not an absolute prerequisite for the determination of secondary structure from circular dichroism spectra: a new scaling method. Anal Biochem.

[24] Bargieri DY, Rosa DS, Braga CJM, Carvalho BO, Costa FTM, Espíndola NM (2008). New malaria vaccine candidates based on the *Plasmodium vivax* merozoite surface protein-1 and the TLR-5 agonist *Salmonella typhimurium* FliC flagellin. Vaccine.

[25] de Alencar BCG, Persechini PM, Haolla FA, de Oliveira G, Silverio JC, Lannes-Vieira J (2009). Perforin and gamma interferon expression are required for CD4+ and CD8+ T-cell-dependent protective immunity against a human parasite, *Trypanosoma cruzi*, elicited by heterologous plasmid DNA prime-recombinant adenovirus 5 boost vaccination. Infect Immun.

[26] Rodriguez M, Alhassan A, Ord RL, Cursino-Santos JR, Singh M, Gray J (2014). Identification and characterization of the RouenBd1987 *Babesia divergens* Rhopty-associated protein 1. PLoS One.

[27] Terkawi MA, Ratthanophart J, Salama A, AbouLaila M, Asada M, Ueno A (2013). Molecular characterization of a new *Babesia bovis* thrombospondin-related anonymous protein (BbTRAP2). PLoS One.

[28] Salama AA, Terkawi MA, Kawai S, Aboulaila M, Nayel M, Mousa A (2013). Specific antibody to a conserved region of *Babesia* apical membrane antigen-1 inhibited the invasion of *B. bovis* into the erythrocyte. Exp Parasitol.

[29] Norimine J, Suarez CE, McElwain TF, Florin-Christensen M, Brown WC (2002). Immunodominant epitopes in *Babesia bovis* rhoptry-associated protein 1 that elicit memory CD4(+)-T-lymphocyte responses in *B. bovis*-immune individuals are located in the amino-terminal domain. Infect Immun.

