# Supplementary figures and images for: A recombinant multi-antigen vaccine formulation containing Babesia bovis merozoite surface antigens MSA-2a1, MSA-2b and MSA-2c elicits invasion-inhibitory antibodies and IFN-γ producing cells
Source: Parasit Vectors. 2016 Nov 14;9:577. doi: 10.1186/s13071-016-1862-1 (PMC5109680; doi:10.1186/s13071-016-1862-1)

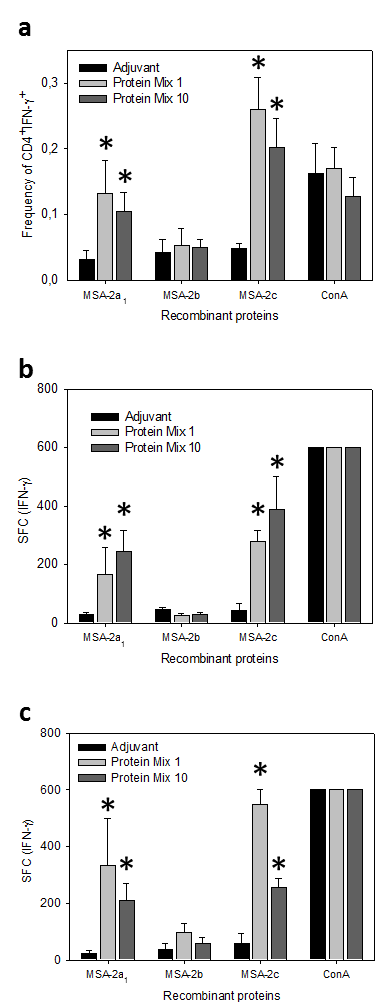

Supplement: Additional file 1: Figure S1. — Cell-mediated immunity of mice immunized with recombinant MSAs. C57BL/6 mice were immunized as described in Fig. 7. Splenic or lymph node cells of these mice were cultured in the presence or absence of the recombinant proteins or ConA, as indicated. a The lymph node cells of these mice were cultured in the presence of anti-CD28, monensin and brefeldin-A with or without the recombinant proteins or ConA, as indicated. After 12 h, cells were stained with anti-CD4 and anti-IFN-γ. The results are expressed as the mean ± SE of the total frequency (%) of CD4+ IFN-γ + cells from 4 mice. b, c After 48 h, IFN-γ secreting cells (spot forming cells, SFC) were estimated by ELISPOT assay. The results are representative of two experiments and are expressed as number of SFC per 5 × 10^5^ splenic (b) or lymph node (c) cells obtained from 4 mice. Asterisks denote statistically significant (b: P = 0.002 for rMSA-2a1; P < 0.001 for rMSA-2c; and c: P < 0.001 for rMSA-2a1; P < 0.001 for rMSA-2c) higher frequencies of number of cells from mice immunized with the mixture of proteins compared to cells from mice injected with adjuvant only. (TIF 49 kb) [file 13071_2016_1862_MOESM1_ESM.tif]
